# Tyrosine kinase LYN is an oncotarget in human cervical cancer: A quantitative proteomic based study

**DOI:** 10.18632/oncotarget.12258

**Published:** 2016-09-26

**Authors:** Shuaibin Liu, Xiaoming Hao, Xiaolan Ouyang, Xiaojing Dong, Yixuan Yang, Tinghe Yu, Jianguo Hu, Lina Hu

**Affiliations:** ^1^ Department of Obstetrics and Gynecology, The Second Affiliated Hospital of Chongqing Medical University, Chongqing, PR China; ^2^ Department of Infectious Diseases, The Second Affiliated Hospital of Chongqing Medical University, Chongqing, PR China

**Keywords:** iTRAQ, cervical cancer, LYN, STAT3, IL-6

## Abstract

Cervical cancer is one of the most common malignant tumor in women. The mechanisms of cervical cancer are intricate and have not been fully understood. Therefore, we employed iTRAQ to obtain novel proteins profile which participates in the tumor oncogenesis of cervical cancer. 3300 proteins were identified aberrantly expressed in cervical cancer, and western bolt was performed to validate the results of iTRAQ. Then, we selected LYN for further study. Immunohistochemistry identified that LYN expression was significantly increased in cervical cancer tissues than that in cancer adjacent normal cervical tissues and normal cervical tissues. The increased LYN expression was significantly correlated with cancer differentiation and FIGO stage. Silencing LYN inhibited cell proliferation, migration and invasion, conversely, overexpression LYN promoted cell proliferation, migration and invasion. In terms of mechanism, LYN could also promote cervical cancer cells metastasis through activating IL-6/STAT3 pathway. *In vivo* study, overexpression LYN promoted tumor growth, meanwhile knockdown LYN inhibited tumor growth. These results indicate that LYN tyrosine kinase is an oncogenic gene and can serve as a novel target for cervical cancer research and therapy.

## INTRODUCTION

Cervical cancer is the third most common female cancer worldwide. It was estimated that there were 527624 new cases of cervical cancer and 265653 deaths from this disease in 2012 [1]. Although Human papillomavirus (HPV) infection is common in young sexually active females, only a small portion of females develop CIN, and even fewer females develop invasive cancer [2], indicating that other factors contribute to the progression to cervical cancer.

Recently, an ultrasensitive and high-throughput proteomic technology which utilizes isobaric tags for relative and absolute quantitation (iTRAQ) coupled with 2-dimensional liquid chromatography and tandem mass spectrometry (MS/MS) analysis has been used to study biomarkers for breast, colon, lung, prostate, stomach, and esophageal [3]. This technology employs a 4-plex set of amine reactive isobaric tags to derivatize peptides at the N-terminus and the lysine side chains, thereby labeling all peptides in a digest mixture [4]. In MS, peptides labeled with any of the isotopic tags are indistinguishable. Upon fragmentation in MS/MS, signature ions are produced and every peptide ion selected for fragmentation generates sequence and abundance data for proteins across up to eight samples, due to the multiplex reagent design [5]. Proteome comparisons were successfully demonstrated in human thinPrep cervical smear using the iTRAQ Mass-Tagging and 2D LC-FT-Orbitrpa-MS [6]. To date, the proteomic analysis of the whole cervical cancer and normal cervical cancer remains largely unexplored. In this study, iTRAQ labeling coupled with high resolution mass spectrometry was carried out to detect the difference proteins between cervical cancer and non-cancer samples. We tried to find some novel proteins associated with cervical cancer which could serve as potential targets for diagnosis of future treatment regimens.

## RESULTS

### iTRAQ identification and quantification of aberrantly expressed proteins

To identify potential biomarkers for cervical cancer, iTRAQ quantification was performed from pooled tissue sample of 7 randomly selected individuals from each of two groups: cervical cancer tissues and non-tumor tissues. Tandem mass spectrometry following immune depletion of albumin and IgG was used to profile the samples.

**Figure 1:**
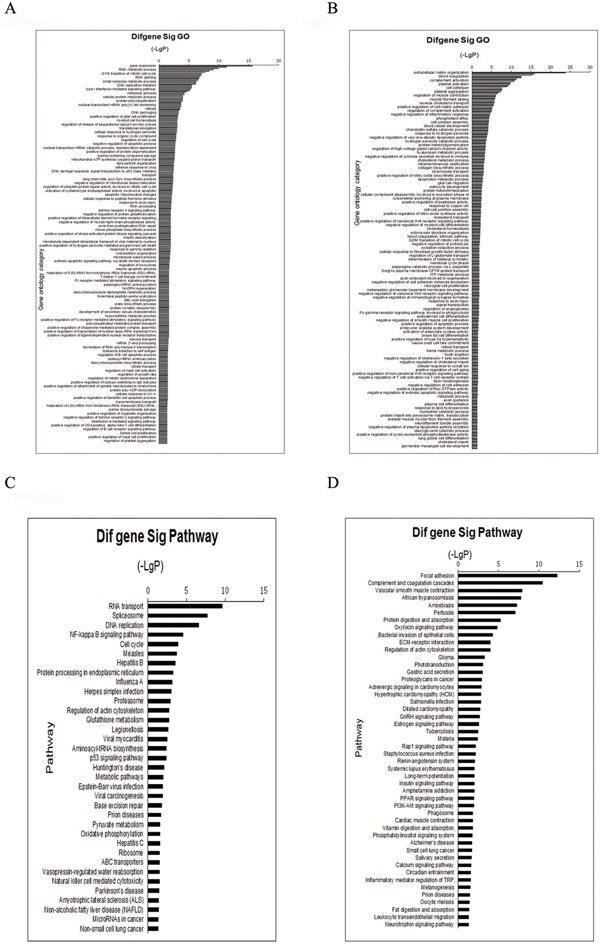
GO and Pathway analyze the proteins identified through iTRAQ proteomics Function identified by iTRAQ categorized. **A.** up-regulated proteins, **B.** down-regulated protein. Pathway identified by iTRAQ categorized: **C.** up-regulated proteins, **D.** down-regulated proteins. Fisher's exact test and test were used to classify the GO category and select the significant pathway, and the threshold of significance was defined by P-value and FDR.

In total, 3300 proteins were identified. For protein identification, a threshold ProtScore value of more than 1.3 was used to attain to 95% confidence level. For subsequent relative quantification analysis, an additional >1.3 or <0.77-fold cutoff (*P* <0.05) was applied to all iTRAQ ratios to minimize false positives when identifying proteins as up-regulated or down-regulated [7, 8]. The strategy resulted in the identification of 330 unique proteins, including 137 up-regulated and 193 down-regulated proteins. Supplementary Table S1 show the top15 up-regulated and 15 down-regulated proteins. Then we used GO-Analysis and Pathway-Analysis to analyze the main function of the differential expression proteins associated with cervical cancer oncogenesis and the related signaling pathways (Figure 1).

### Validation of altered expression levels of proteins

**Figure 2:**
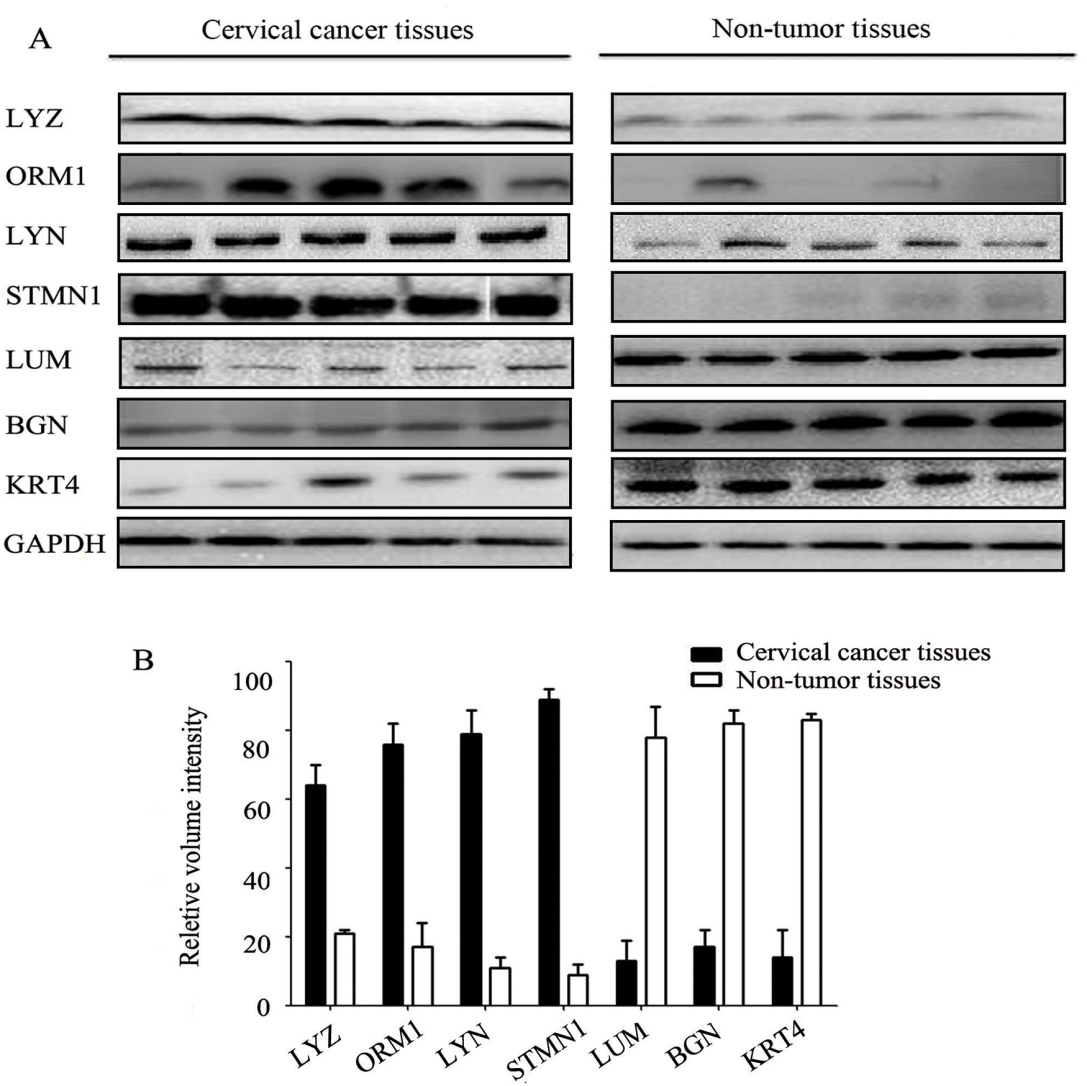
Western blot analysis of differentially expressed proteins in cervical cancer tissues and non-tumor tissues Error bars represent standard error.

Western blotting was utilized to measure the levels of the proteins to validate the results of iTRAQ (Figure 2). Western blot analysis confirmed that LYZ, ORM1, LYN, STMN1 significantly increased in cervical cancer, while LUM, BGN, KRT4 significantly decreased. This trend matched what was observed in the iTRAQ method. LYN is a member of SRC family of protein tyrosine kinases, and is the key regulators of several cellular processes as well, including cancer cell growth, migration, invasion, and survival [9, 10]. However, little is known about the relationship between LYN and cervical cancer, as well as the cellular function of LYN in cervical cancer.

### The expression of LYN in cervical cancer tissues and normal cervical tissues

**Figure 3:**
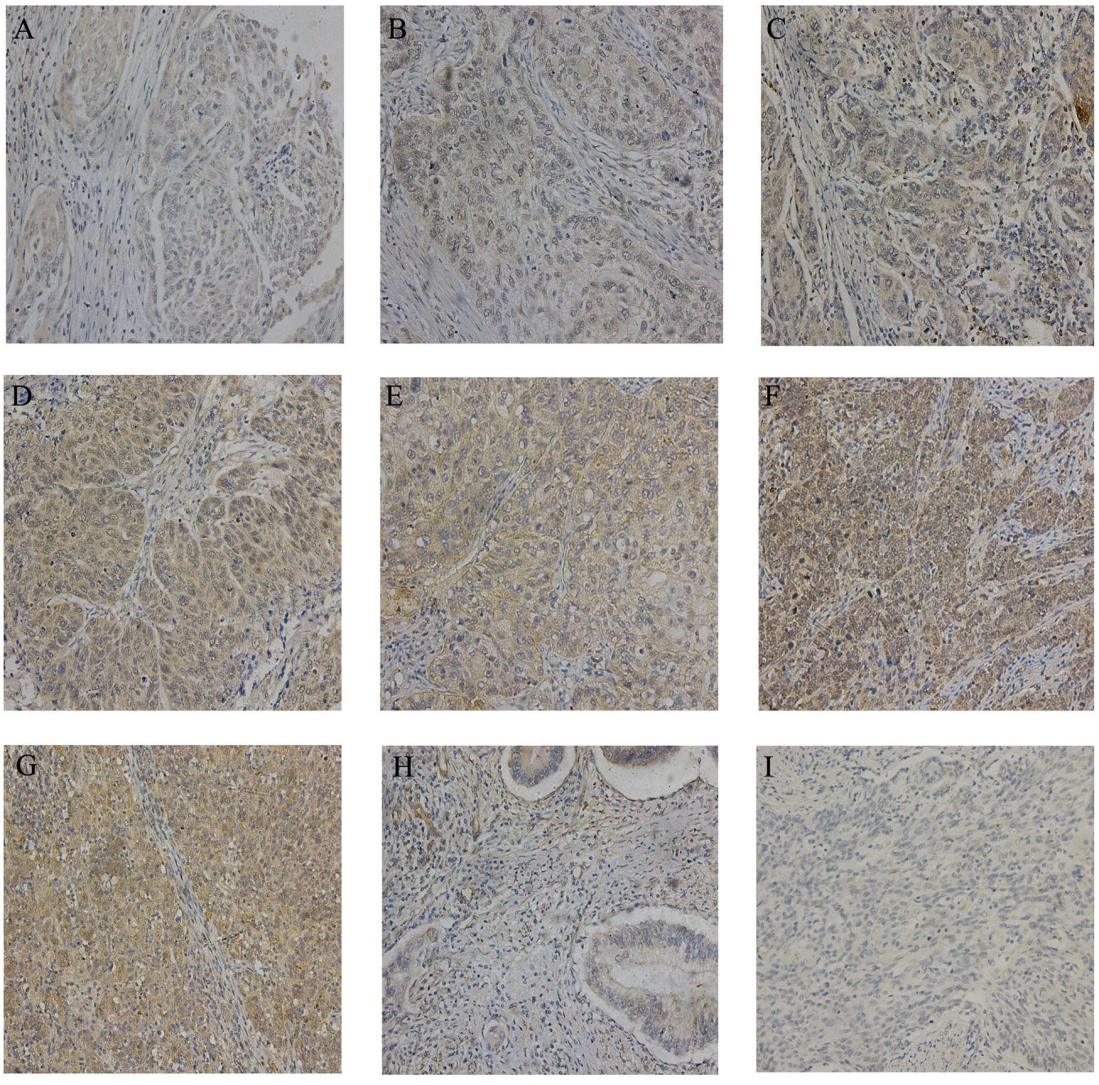
Representative IHC images of LYN in tissue microarrays The expression of LYN in different stage of cervical cancer samples A-G. **(A).** stage IA; **(B).** stage IB; **(C).** stage IC; **(D).** stage IIA. **(E).** stage IIB; **(F).** stage III; **(G).** stage IV; **(H).** cancer adjacent normal cervical tissues; **I.** normal cervical tissue. Original magnification, 200X.

IHC evaluation of LYN was performed on a commercial tissue microarray containing 192 cervical cancer tissues, 15 cancer adjacent normal cervical tissues and 1 normal cervical tissue. The age of 122 cases were less than 50 years and 86 cases were equal or greater than 50 years. The expression of LYN significantly increased in cervical cancer tissues than that in cancer adjacent normal cervical tissues and normal cervical tissue (*P* <0.05, Figure 3, Table 1). According to FIGO staging system, 153 samples were classified as stage I, 34 as stage II, 5 as stage III and stage IV. There were 180 cases of squamous cell carcinoma, 11 cases of adenosquamous carcinoma and 1 case of adenocarcinoma in the microarray. IHC results showed that high LYN expression in samples had positive correlation with FIGO stage (*P* <0.05) and tumor grade, but obvious relation was found neither with the patient age (*P* >0.05) nor with tumor type (squamous cell carcinoma versus adenosquamous carcinoma, *P* >0.05. There was only one case in adenocarcinoma group, so we did not include it in the calculation).

**Table 1:** Relationship between expression of LYN and clinic pathology in 208 samples

Characteristics	No. of samples (n=208)	LYN expression		P value
		Low no. (%)	High no. (%)	
Age (years)				
<50	122	65(53.3)	57(46.7)	0.892
≥50	86	45(52.3)	41(47.7)	
Tissue type Normal cervical	1	1(100.0)	0(0.0)	0.180
Cancer adjacent normal cervix tissue	15	11(73.3)	4(26.7)	
Cancer tissues	192	102(53.1)	90(46.9)	
FIGO stage				<0.001
I/IA/IB/IC	153	95(62.1)	58(37.9)	
II/IIA/IIB	34	7(20.6)	27(79.4)	
III/IIIA/IIIB/IV	5	0(0.0)	5(100.0)	
Grade				
1	31	25(80.6)	6(19.4)	0.001
2	110	57(51.8)	53(48.2)	
3	51	20(39.2)	31(60.8)	
Tumor type				0.872
Squamous cell carcinoma	180	96(53.3)	84(46.7)	
Adenosquamous carcinoma	11	6(54.5)	5(45.5)	
Adenocarcinoma	1	0(0.0)	1(100.0)	

### LYN regulates cellular proliferation

**Figure 4:**
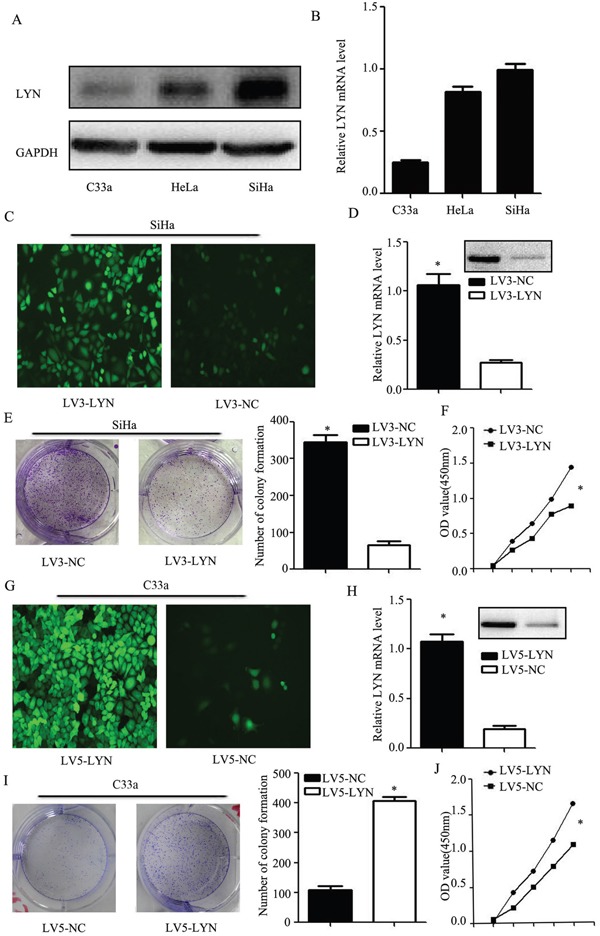
LYN regulates cellular proliferation of cervical cancer cell **A.** Western blot analysis of LYN expression in HeLa, SiHa and C33a cell lines. **B.** Relative expression of LYN mRNA in HeLa, SiHa and C33a cell lines. **C.** Transfection efficiency after LV3-LYN transfected SiHa cells. **D.** After infected with LV3-LYN, the protein and mRNA level were significantly decreased. **E, F.** Colony formation assay and CCK-8 were used to detect SiHa cell proliferation. **G.** Transfection efficiency after LV5-LYN transfected C33a cells. **H.** After infected with LV5-LYN, the protein and mRNA level were significantly increased. **I, J.** Cell proliferation was detected by colony formation assay and CCK-8 in C33a cells. Error bars represent standard error. * *p* < 0.05.

Compared with HeLa and SiHa cell lines, mRNA and protein level of LYN expression in the C33a cell line was the lowest. Therefore, C33a cell line was selected out for the further exogenous expression research. Compared with C33a and HeLa cell lines, mRNA and protein level of LYN expression in the SiHa cell line was the highest (Figure 4A, 4B, Supplementary Figure S1A). So, SiHa cell line was selected for knockdown research. GFP fluorescent image was used to verify the transfection efficiency (Figure 4C, 4G). The mRNA and protein level of LYN decreased significantly in LV3-LYN group than that in LV3-NC group (Figure 4D, Supplementary Figure S1B), while it increased significantly in LV5-LYN group than that in LV5-NC group (Figure 4H, Supplementary Figure S1C).

Our data showed that the ability of the cell proliferation had decreased more remarkably in LV3-LYN group than that in the LV3-NC group (*P* <0.05; Figure 4E, 4F). Compared with the LV5-NC group, cell proliferation of LV5-LYN group significantly increased (*P* <0.05; Figure 4I, 4J).

### The effect of LYN on cervical cell migration and invasion

**Figure 5:**
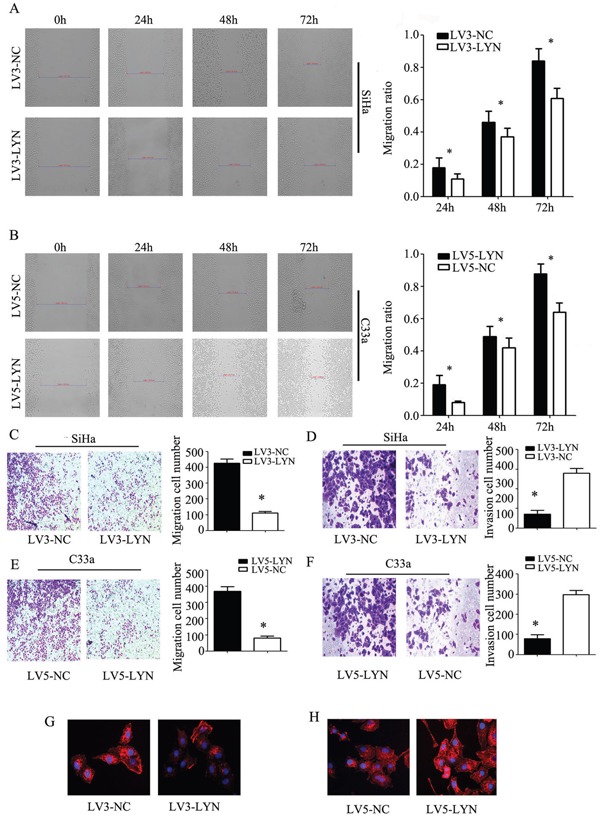
The effect of LYN on cell migration and invasion **A, B.** Wound healing assay was used to detect the migration ability of cervical cancer SiHa and C33a cells. **C, D.** Transwell migration and matrigel invasion assays were used to detect the invasion ability in SiHa cells transfected with LV3-LYN and LV3-NC. **E, F.** Transwell migration and matrigel invasion assays were used to detect the invasion ability in C33a cells transfected with LV5-LYN and LV3-NC. (G, H) Phalloidin was used to label the F-actin. Original magnification, 400X. Error bars represent standard error. * *p* < 0.05.

We employed transwell migration assay and wound healing assay to detect the cell migration capability and use Matrigel invasion assays to detect the cell invasion capability. Knockdown of LYN resulted in the inhibition of the migration and invasion ability of SiHa cell line (Figure 5A, 5C, 5D). On the other hand, when we infected the C33a cells with LV5-LYN to overexpress LYN, we found that the migration and invasion capabilities of C33a were increased remarkably (Figure 5B, 5E, 5F).

The first step of cancer metastasis is invasion in the surrounding tissues. And, the key point of the celluar invasion and migration is cytoskeletal reorganization. In Ekaryotic cells, cell migration requires the formation of cell membrane extensions containing actin filaments [11]. On this basis, we use phalloidin to label the F-actin. Interestingly, we found that F-actin staining was chiefly concentrated at edge of SiHa and C33a cell lines. After infecting LV3-LYN, F-actin staining of SiHa cell line significantly decreased and the formation of membrane ruffles were prevented (Figure 5G). Compared with LV5-NC group, the amount of F-actin staining increased and the formation of membrane ruffles could be seen in LV5-LYN group (Figure 5H). So, we deduced that LYN could regulate cell migration and invasion by induced F-actin remodeling.

### LYN binds to P-STAT3 in cervical cancer cells

**Figure 6:**
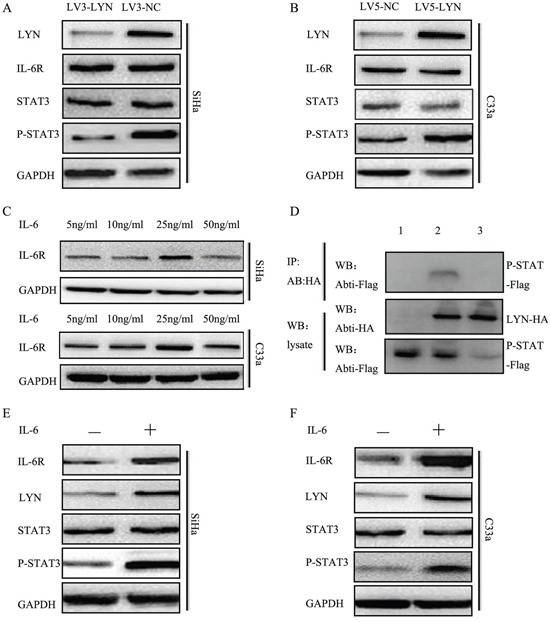
LYN promoted cervical cancer cells metastasis through activating IL-6/STAT3 pathway **A.** The protein level of LYN, IL-6R, STAT3, P-STAT3 was detected after transfected with LV3-LYN and LV3-NC. **B.** The protein level of LYN, IL-6R, STAT3, P-STAT3 was detected after transfected with LV5-LYN and LV5-NC. **C.** The optimal concentration of IL-6 in SiHa and C33a cells. **D.** Co-immunoprecipitation demonstrated LYN binds to P-STAT3. **E, F.** The protein level of LYN, IL-6R, STAT3, P-STAT3 after treated SiHa and C33a cells with IL-6 (25 ng/ml) for 48 hours.

In order to understand which protein interacted with LYN, we applied BioGRID3.4. We found that STAT3 could directly interact with LYN. Therefore, we designed co-immunoprecipitation to validate the direct relationship between LYN and P-STAT3 (Figure 6D). P-STAT3-Flag and LYN-HA were co-transfected into SiHa cells and samples were collected after 24h. Then, Anti-HA Tag Antibody was used to pull the other interact proteins and detected by Anti-Flag Tag Antibody. We found that band was detected when the SiHa cells were co-transfected with P-STAT3-Flag and LYN-HA (lane 2). But the band could not be detected when transfected with P-STAT3-Flag (lane 1) or LYN-HA (lane 3). These findings revealed that LYN may act in combination with P-STAT3 in cervical cancer cells.

### LYN promoted cervical cancer cells metastasis through activating IL-6/STAT3 pathway

Our data showed that P-STAT3 expression significantly decreased when we transfected SiHa cells with LV3-LYN (Figure 6A, Supplementary Figure S1D). On the other hand, when we treated C33a cells with LV5-LYN, P-STAT3 expression significantly increased (Figure 6B, Supplementary Figure S1E). But when we knockdown or overexpression LYN protein, there were no significant change on the expression of IL-6R and STAT3. In order to determine whether IL-6 could regulate LYN expression in cervical cancer cell lines, we analyzed LYN expression in SiHa and C33a cells after IL-6 treatment. Cervical cancer SiHa and C33a cells were treated with IL-6 (5, 10, 25, 50 ng/ml) for 48 hours. Compared with other concentration, protein level of P-STAT3 significantly increased at 25 ng/ml (Figure 6C, Supplementary Figure S1F-S1G). Then we detected protein level of LYN, IL-6R, STAT3, P-STAT3 after treated with IL-6 (25 ng/ml) for 48 hours. We found that the expression of LYN, IL-6R and P-STAT3 significantly increased (Figure 6E, 6F, Supplementary Figure S1H-S1I). So, we demonstrated that down-regulated and up-regulated LYN expression blocked and activated signaling via IL-6/STAT3 pathway.

### *In vivo* tumor xenograft study

**Figure 7:**
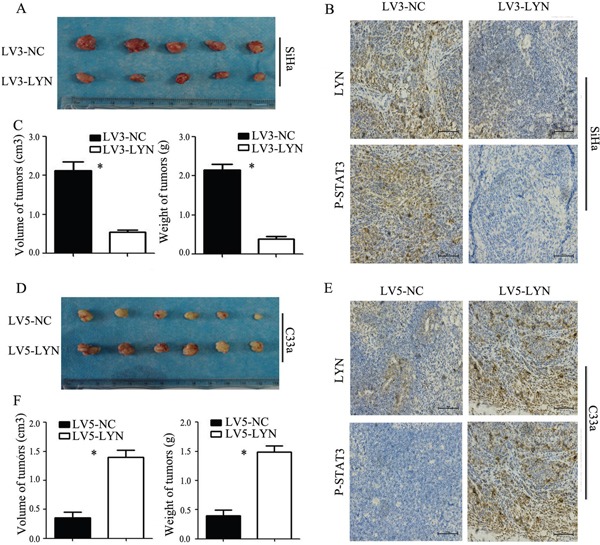
*In vivo* tumor xenograft study **A, C.** Average tumor volume and weight on day 21 after tumor cell injected with LV3-LYN and LV3-NC. **B.** Representative microphotographs of immunohistochemical analysis the expression of LYN and P-STAT3 on tumor xenografts. **D, F.** Average tumor volume and weight on day 21 after tumor cell injected with LV5-LYN and LV5-NC. **E.** Representative microphotographs of immunohistochemical analysis the expression of LYN and P-STAT3 on tumor xenografts. Original magnification, 200X. Error bars represent standard error. * *p* < 0.05.

Xenograft tumorigenesis in nude mice was used to explore the effect of LYN on tumor formation of cervical cancer. The LV3-LYN SiHa cells and LV3-NC SiHa cells were implanted subcutaneously into the right armpit of nude female mice. After 21 days, we got the tumors from mice. Compared with LV3-NC group, the average volume and weight of tumor in LV3-LYN group were significantly smaller and lighter (*P*<0.05; Figure 7A, 7C). IHC revealed that expression of LYN and P-STAT3 in LV3-LYN group was decreased than that in the LV3-NC group (*P*<0.05; Figure 7B). These data showed that silencing LYN could block tumor formation *in vivo* and inhibit the expression of P-STAT3 *in vivo*. On the other hand, the average volume and weight of tumor were increased more significantly in LV5-LYN group than those in LV5-NC group (*P* <0.05; Figure 7D, 7F). IHC showed that the expression of LYN and P-STAT3 in LV5-LYN group was increased than that in the LV5-NC group (*P* <0.05; Figure 7E). These data showed that overexpression LYN could promote tumor formation and the expression of P-STAT3 *in vivo*.

## DISCUSSION

Despite the great success of early screening programs of cervical cancer, cervical cancer is still the leading cause of gynecological death among women worldwide [12]. Accounting for 80-90% of cervical cancer, cervical squamous cell carcinoma is one of the most frequent types of cervical cancers. Studies show that more than 99% of patients with cervical squamous cell carcinoma is persistent human papilloma virus (HPV) infection [13]. But the virus alone is not enough to develop cancer. The mechanisms of cervical cancer initiation, infiltration and metastasis involving many protein have not yet been fully elucidated. Therefore, novel proteins are necessary in understanding the procedure of progression and metastasis of cervical cancer.

In our study, the iTRAQ proteomics approach was used to identify aberrantly expressed proteins in tumor and non-tumor cervical tissues. Then, western blot assays were used to validate the results of iTRAQ. We found that LYZ, ORM1, LYN and STMN1 significantly increased in cervical cancer samples. On the other hand, LUM, BGN and KRT4 significantly decreased in cervical cancer samples. LYN was selected as the target in the following study. In addition, this study demonstrates the iTRAQ proteomics approach for high throughput protein quantification is credible and some novel proteins discovered here may be employed as potential markers for cervical cancer diagnosis and treatment.

LYN, a member of non-receptor protein tyrosine kinases (PTKs), is located at chromosome 8q13. LYN establishes thresholds by acting as both a positive and negative modulator of a variety of signaling responses [14]. In addition, LYN overexpression has been reported in several cancers, such as chronic myelogenous leukemia [15], colorectal cancer [16], breast cancer [17], prostate cancer [18], oral cancer [19], renal cancer [20] and gastric cancer [21]. Nonetheless, little study has evaluated the role of LYN in cervical cancer.

In our research, we found that the expression of LYN was significantly higher in cervical cancer tissues than that in cancer adjacent normal cervical tissues and normal cervical tissue. We also found that high LYN expression in samples had positive correlation with FIGO stage and tumor grade. These results showed that LYN is an oncogene in cervical cancer. LYN may play an important role in tumorigenesis of cervical cancer.

In order to investigate the function of LYN in cervical cancer, we employed lentiviral vector to study the functional assays of LYN knockdown and overexpression. We found that down-regulated or up-regulated LYN could inhibit or promote cervical cancer cells migration, invasion and cell proliferation *in vitro* and *vivo*. So our results demonstrated that LYN could promote metastasis of cervical cancer. And our further research demonstrated that the knockdown or overexpression of LYN could induce F-actin remodeling. Our results indicated that LYN may play an important role in the tumor metastasis process.

STAT proteins, especially STAT3, are crucial for both the extrinsic and the intrinsic pathways underlying cancer inflammation. Persistent activation of STAT3, in diverse human cancers, increases tumor cell proliferation, survival, angiogenesis and metastasis [22]. Also, constitutively activated STAT3 has been detected in cervical cancer cells [23]. Then, we designed a co-immunoprecipitation to validate the relationship between LYN and P-STAT3. We found that LYN could bind to P-STAT3 in cervical cancer cells.

IL-6 is a central proinflammatory cytokine involved in female genital infection and is abundant in the microenvironment of cervical cancer [24, 25]. Various researches have suggested that IL-6 is important in cervical carcinogenesis [26]. Meanwhile, IL-6 is one of the key activators of STAT3, so we treated it to cervical cancer cell lines to reveal the relationship among IL-6R/LYN/STAT3. We found that down-regulation of LYN in SiHa cells decreased the expression of P-STAT3. But the expression of IL-6R had no significant change. Up-regulated of LYN in C33a cells increased the expression of P-STAT3. These results revealed that LYN could regulate P-STAT3, but could not regulate IL-6R. We up-regulated the expression of IL-6R by treating SiHa and C33a cells with IL-6, which leading to the expression of LYN and P-STAT3 increased. These results showed that IL-6 could affect the expression of LYN and P-STAT3. All these results indicated that LYN promoted cervical cancer cells metastasis through activating IL-6/STAT3 pathway.

In summary, the results herein reported highlight the iTRAQ proteomics approach for high throughput protein quantification is credible and some novel proteins discovered here may serve as potential markers and LYN tyrosine kinase can serve as a novel target for cervical cancer research and therapy.

## MATERIALS AND METHODS

### Reagents

Seven-plex iTRAQ reagent kits were obtained from Applied Biosystems (Foster City, CA). Rabbit monoclonal antibodies against Cytokeratin 4 (KRT4, ab51599), Biglycan (BGN, ab109369), Lysozyme (LYZ, ab108508), Lumican (LUM, ab168348), Stathmin 1 (STMN1, ab52630), Mitofusin 2 (MFN 2, ab124773) were purchased from Abcam. Rabbit monoclonal antibodies against LYN (2796) was purchase from CST (LYN Rabbit mAb detects endogenous levels of total LYN protein. This antibody does not cross-react with any other Src-family members.).

### Tissue specimens

Seven human cervical cancer samples and seven paired non-cancer samples were collected at the Second Affiliated Hospital of Chongqing Medical University. The tissue microarray slides containing malignant and normal cervical tissues (n=208) was purchased from US Biomax, Inc. (Rockville, MD). The experiments were reviewed and approved by the research ethics committee of Chongqing Medical University.

### Protein extraction and iTRAQ labeling

Total protein was extracted from the human cervical cancer samples and non-cancer samples. 2-D Quant kits (Amersham Biosciences, Little Chalfont, UK) were used to determine the total concentrations. For each pool, 100 μg of protein were precipitated, denatured, cysteine blocked and digested with sequencing grade modified trypsin [3]. The labeled samples were mixed before analysis. Then samples were labled using the iTRAQ reagents (pooled non-tumor samples, 113 tags; pooled tumor samples, 114, 115 tags).

### Peptides fractionation

The iTRAQ-labeled peptides were fractionated by immobilized pH gradient isoelectric focusing (IPG-IEF). After dissolution in Pharmalyte and urea (Amersham Biosciences) solution, the samples were applied on pH 3-10 IPG strips (Amersham Biosciences) and focused with an IPGphor isoelectric focusing system (Amersham Biosciences). Peptides were extracted from the gel using a formic acid/ACN solution and fractions lyophilized and purified (SPE C18 column). The purified fractions were lyophilized before analysis with mass spectrometric.

### Mass spectrometry

Mass spectrometric analysis was performed using a nano-LC coupled online to QStarXL mass spectrometer (Applied Biosystems). The mass spectrometer was set to perform information-dependent acquisition (IDA) in the positive ion mode at a mass range of 300-1800 m/z. Peptides with +2 to +4charge states were selected for tandem mass spectrometry, and the time of summation of MS/MS events was set to 3s. The two most abundantly charged peptides above a 10 count threshold were selected for MS/MS and dynamically excluded for 60s with ±50 mDa mass tolerance.

### Database analysis

Data were processed with ProteinPilot v4.5 (Applied Biosystems). Each MS/MS spectrum was searched against the UniProt database. A threshold of confidence >99% and a local false discovery rate of <1% were used for both protein identification and quantitative analysis. Protein identification was accepted based on ProteinPilot confidence scores. P-values were required to be <0.05 for relative quantification by iTRAQ. The PeakView 1.1 software was used to extract ion chromatograms.

### Cell lines

Human cervical cancer cell lines HeLa, SiHa and C33a were purchased from China Center for Type Culture Collection (Wuhan, China). All cells were cultured Rosewall Park Memorial Institute 1640 medium (Gibco, San Diego, CA), containing 10% fetal bovine serum(Gibco, San Diego, CA) and 1% Penicillin & Streptomycin solution. All human cervical cancer cell lines were maintained at 37°C with 5.0% carbon dioxide.

### Immunohistochemistry (IHC)

IHC evaluation of LYN was performed on the tissue microarray. IHC was preformed according to the Streptavidin/Peroxidase kit instructions (SPlink Detection Kits, ZSGB-BIO, China). After deparaffinized and rehydrated, the sections were subjected to heat in citrate buffer –induced antigen retrieval for 15 min. Endogenous peroxidase activity was quenched with 3% H_2_O_2_ for 15 min. Thereafter, sections were blocked with goat serum for 15 min. Anti-LYN (1:100) was incubated overnight at 4°C. Then sections were incubated with HRP-conjugated secondary antibodies for 10 min and incubated in horseradish enzyme-labeled chain avidin solution for 10 min at 37°C and reacted with a DAB Horseradish Peroxidase Color Development Kit and counterstained with hematoxylin. Staining intensity was graded on a 0–3 scale as follows: 0 (absence of staining), 1 (weakly stained), 2 (moderately stained), and 3 (strongly stained). The percentage of positive tumor cells was scored as follows: 0 (absence of tumor cells), 1 (<33% tumor cells), 2 (33–66% tumor cells) and 3 (>66% tumor cells). Immunohistochemical score (ranging from 0 to 9) was calculated by multiplying the intensity score and the percentage score [27]. The same qualified pathologist analyzed all the IHC data to ensure consistency.

### Quantitative real-time polymerase chain reaction (PCR)

Total RNA was isolated from all cancer cells using TRIzol reagent (Ambion Inc., Austin, TX, USA) according to the manufacture's protocol. Then RNA was quantified by using nanodrop spectrophotometer. cDNA was synthesized using the Rever Tra Ace qPCR RT kit (TOYOBO, FSQ-101, Japan). The primers used for amplifying LYN and GAPDH were synthesized by GeneCopoeia Inc. The real-time PCR kit was purchased from Kapa Biosystems Inc. (Boston, US). Each sample was analyzed in triplicates. Quantification of genes transcription were determined with the method of 2^−ΔΔCT^ [28]. RT-PCR analysis was repeated at least three times.

### Western blotting analysis

Total protein extracted from each tissue sample was separated in 10% polyacrylamide gel, and electrotransferred to polyvinylidene fluoride membranes (Millipore Corporation, Billerica, MA). The bands were blocked in 5% dried milk for 2h at room temperature. Primary antibodies (1:1000-1:2000) against LYZ, ORM1, LYN, STMN1, LYM, BGN, KRT4 were incubated overnight. After washing with Tris-Buffered Saline (TBS) containing 0.1% Tween-20 (TBS-T), the membranes were further incubated with an anti-rabbit IgG antibody conjugated with horseradish peroxidase (ZSGB-BIO, China) for 1h. After washing with TBS-T, the membranes were detected with an ECL detection system (KeyGen Biotech Inc, Nanjing, China). All of the Western blot analyses were repeated at least three times.

### Transfections

Three LYN-targeted small interfering RNA (siRNA) constructs and control NC siRNA were synthesized by Gnenpharma Co., Ltd. (Shanghai, China). The details of the target sequence for LYN-728 (5’-GCUGGAGCUUUCCUUAUUATT-3’), LYN-894 (5’-GCGACAUGAUUAAACAUUATT-3’), LYN-1484 (5’-GGUGCUAAGUUCCCUAUUATT-3’) were obtained and used for the gene silencing studies. Control NC siRNA scrambled target sequence (5’-UUCUUCGAAC GUGUCACGUTT-3’) was used as control. Lentiviral vector expressing shRNA targeting LYN (named LV3- LYN) was provided by Genepharma Co., Ltd. (Shanghai, China). LYN -lentiviral expression vector (named LV5- LYN) was provided by Genechem Co., Ltd (Shanghai, China).

### Colony forming assay

SiHa cells infected with LV3-LYN and C33a cells infected with LV5-LYN were cultured by seeding 1000 cells in 6-well plates. All cells were incubated at 37°C with 5.0% carbon dioxide for 14 days. After 14 days, the number of colonies formed was checked. All experiments were triplicated.

### Proliferation assay

SiHa cells and C33a cells were seeded into 96-well plates at a density of 2000 cells per well and then transfected with LV3-LYN, LV3-NC and LV5-LYN, LV5-NC. Cell proliferation was tested using a CCK-8 Kit (DNDOJAN, Japan) every 24 h after transfection for 7 days (the reactions were incubated for 1 h at 37°C and 5% CO_2_; detection: 450 nm, reference: 630 nm). The experiment was repeated three times.

### Wound healing assay

Migration of SiHa and C33a cells were analyzed using the wound-healing assay *in vitro*. SiHa cells infected with LV3- LYN and C33a cells infected with LV5- LYN were cultured in 6-well plates and cultivated until 90% growth confluence. Wounds were incised the monolayer cells with a sterile pipette tip. At 0, 24, 48 and 72 hours after the wounding, cells were observed under the light microscope. The distance between the two wounds were measured at each time point and expressed as the average percent of wound closure as compared to that at zero time. The experiment was repeated three times.

### Transwell membrane based migration and invasion assay

The effect of LYN knockdown and overexpression on the ability of SiHa and C33a cells to migrate through a filter or to invade through a biological barrier was examined by transwell insert chambers having 8-μm pore filters (Corning, New York, NY). For Matregel migration assays, the upper side of an 8 μm pore, 6.5-mm polycarbonate transwell filter (Corning, New York, NY) chamber was uniformly coated with Matrigel basement membrane matrix (BD Biosciences, Bedford, MA) for 2 h at 37°C before the cells were added. 2 × 10^5^ cells were seeded in the upper chambers with 200μl serum-free media, and the lower chambers were filled with 750μl complete media containing fetal bovine serum, which can induce cell migration. After 24h, cells that migrated/invaded to the lower surface of the filter were fixed with 4% paraformaldehyde, stained in 0.5% crystal violet, and counted using a microscope. Each experiment was performed in triplicates and repeated thrice.

### Label the F-actin

Perform formaldehyde fixation. Incubate cells with 3.0-4.0% formaldehyde in PBS at room temperature for 30 minutes. Rinse the fixed cells 2-3 times in PBS. Next, Add 0.1% Triton X-100 in PBS into fixed cells for 5 minutes to increase permeability. Rinse the cells 2-3times in PBS. Add 100ul/well (96-well plate) of phalloidin conjugate working solution into the fixed cell, and stain the cells at room temperature for 60 minutes. Rinse cells gently with PBS 2-3 times to remove excess phalloidin conjugate before plating, sealing and imaging under microscope.

### Immunoprecipitation

Immunoprecipitation analysis was performed using Protein A+G Sepharose Beads (P001-5, Shanghai Yanji Biotechnology) for 3h at 4°C. Briefly, protein A+G Sepharose beads were washed and pre-incubated with primary antibody for 1 hour at 4°C. Cells were lysed with RIPA buffer (Millipore, Billerica, MA) supplemented with phosphatase/protease inhibitor cocktail and 1 mM PMSF through sonication and centrifugation. The proteins were quantified by BCA Protein Assay Reagent Kit (Beyotime, China). Bound protein was eluted off the beads by boiling in sample buffer and was then analyzed by western blot.

### *In vivo* tumor xenograft study

Six-week-old female balb/c nude mice were purchased from the Experimental Animal Center of Chongqing Medical University. The research protocol was approved and mice were maintained in accordance with the institutional guidelines of the Committee on the Use and Care on Animals (Chongqing Medical University, Chongqing, China). Cervical cancer SiHa and C33a cells were infected with indicated lentiviral vectors and injected (5 × 10^6^ cells per mouse in 200 ul) subcutaneously into the left armpit of 6-week-old BALB/c nude mice. 21 days later, animals were sacrificed to confirm the presence of tumors and weigh the established tumors.

### Statistical analysis

All statistical analyses were performed using SPSS software, v 17.0 (Chicago, IL). Comparisons between groups were analyzed using a Student's –t test or a Mann-Whitney U test. The chi-square test was used to compare the associations between LYN overexpression and clinicopathologic variables of cervical cancer and non-tumor samples. All experiment was performed in triplicates. A P-value <0.05 was considered statistically significant.

## SUPPLEMENTARY FIGURE AND TABLE




