# Supplementary material for: Tyrosine kinase LYN is an oncotarget in human cervical cancer: A quantitative proteomic based study
Source: Oncotarget. 2016 Sep 26;7(46):75468–81. doi: 10.18632/oncotarget.12258 (PMC5342753; doi:10.18632/oncotarget.12258)
Supplement: Supplementary file 1 [file oncotarget-07-75468-s001.pdf]

Tyrosine kinase LYN is an oncotarget in human cervical cancer: A quantitative proteomic based study

SUPPLEMENTARY FIGURE AND TABLE

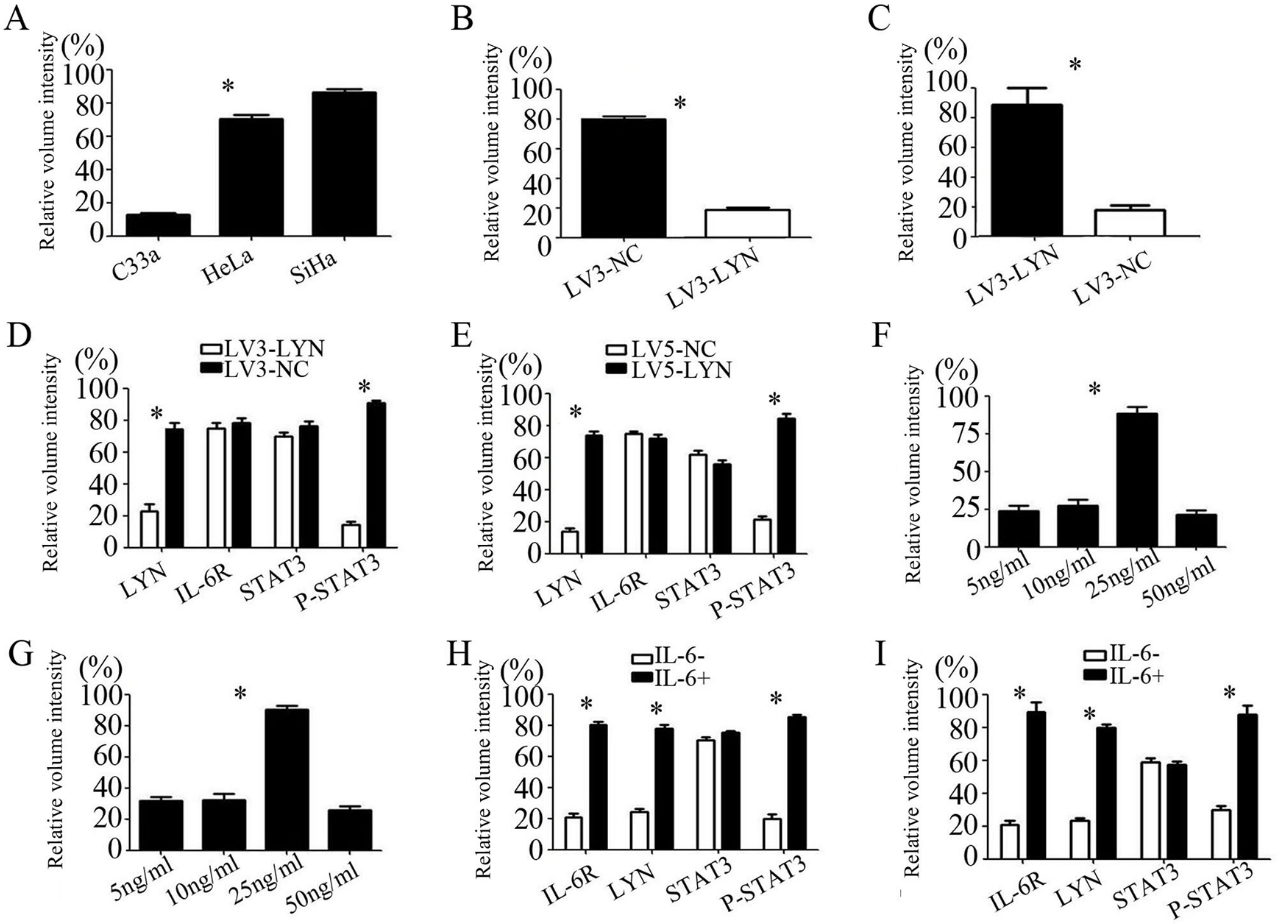

Supplementary Figure S1: Relative volume intensity of Western blot. A. The expression of LYN protein level in different cervical cancers. **B-C.** The protein level of LYN after transfected with LV3-LYN and LV5-LYN. **D-E.** The protein level of LYN, IL-6R, STAT3, P-STAT3 was detected after transfected with LV3-LYN and LV5-LYN. **F-G.** The optimal concentration of IL-6 in SiHa and C33a cells. **H-I.** The protein level of LYN, IL-6R, STAT3, P-STAT3 after treated SiHa and C33a cells with IL-6 (25 ng/ml) for 48 hours.

Supplementary Table S1: Partial list of the differentially expressed proteins identified by iTRAQ analysis between cervical cancer samples and non-cervical cancer samples

See Supplementary File 1
