# Supplementary material for: Tyrosine kinase LYN is an oncotarget in human cervical cancer: A quantitative proteomic based study
Source: Oncotarget. 2016 Sep 26;7(46):75468–81. doi: 10.18632/oncotarget.12258 (PMC5342753; doi:10.18632/oncotarget.12258)
Supplement: Supplementary file 2 [file oncotarget-07-75468-s002.docx]

**Supplemental table 1. Partial list of the differentially expressed proteins identified by iTRAQ analysis between cervical cancer samples and non-cervical cancer samples.**

| **N** | **Accession** | **Protein name** | **Gene_Symbol** | **Peptides (95%)** | **114:113** | **PVal 114:113** | **115:113** | **PVal 115:113** |
| --- | --- | --- | --- | --- | --- | --- | --- | --- |
| 1 | IPI:IPI00019038.1 | LYZ Lysozyme C | LYZ | 3 | 14.454 | 0.152 | 2.3335 | 0.591 |
| 2 | IPI:IPI00941197.1 | PCID2 Uncharacterized protein | PCID2 | 4 | 13.932 | 0.002 | 8.3176 | 0.003 |
| 3 | IPI:IPI00477118.3 | TMEM214 Isoform 1 of Transmembrane protein 214 | TMEM214 | 5 | 12.023 | 0.01 | 3.0479 | 0.198 |
| 4 | IPI:IPI00420084.3 | BID Isoform 2 of BH3-interacting domain death agonist | BID | 3 | 10.864 | 0.117 | 16.444 | 0.143 |
| 5 | IPI:IPI00793989.2 | NCKAP1L nck-associated protein 1-like isoform 2 | NCKAP1L | 4 | 9.4624 | 0.002 | 3.1046 | 0.145 |
| 6 | IPI:IPI00031514.1 | RFC5 Replication factor C subunit 5 | RFC5 | 8 | 8.7902 | 0.024 | 12.134 | 0.007 |
| 7 | IPI:IPI00218646.3 | CYBB Cytochrome b-245 heavy chain | CYBB | 11 | 8.3176 | 0.005 | 2.1281 | 0.918 |
| 8 | IPI:IPI00940936.1 | LYN LYN protein (Fragment) | LYN | 6 | 7.656 | 0.131 | 7.1121 | 0.088 |
| 9 | IPI:IPI00946150.1 | SSR3 cDNA FLJ52061, highly similar to Translocon-associated protein subunit gamma | SSR3 | 5 | 7.5162 | 0.483 | 3.4041 | 0.594 |
| 10 | IPI:IPI00479503.1 | DYNLT1 Uncharacterized protein | DYNLT1 | 3 | 7.379 | 0.402 | 8.3176 | 0.369 |
| 11 | IPI:IPI00843996.1 | SRSF3 cDNA FLJ52832, highly similar to Splicing factor, arginine/serine-rich 3 | SRSF3 | 3 | 7.1779 | 0.139 | 6.3096 | 0.174 |
| 12 | IPI:IPI00020021.3 | DEK Protein DEK | DEK | 9 | 6.9823 | 0.122 | 4.8306 | 0.133 |
| 13 | IPI:IPI00023101.1 | RQCD1 Cell differentiation protein RCD1 homolog | RQCD1 | 4 | 6.6681 | 0.062 | 8.4723 | 0.038 |
| 14 | IPI:IPI00291792.2 | ITGB2 Integrin beta-2 | ITGB2 | 9 | 6.4863 | 0.14 | 3.1915 | 0.238 |
| 15 | IPI:IPI00100656.3 | TECR Isoform 1 of Trans-2,3-enoyl-CoA reductase | TECR | 6 | 6.4863 | 0.067 | 4.9204 | 0.067 |
| 16 | IPI:IPI00921956.1 | SYNM Isoform 3 of Synemin | SYNM | 3 | 0.6546 | 0.479 | 0.3767 | 0.245 |
| 17 | IPI:IPI00296922.4 | LAMB2 Laminin subunit beta-2 | LAMB2 | 24 | 0.6546 | 0.014 | 0.2421 | #### |
| 18 | IPI:IPI00909113.1 | PEX5 cDNA FLJ56404, highly similar to Peroxisomal targeting signal 1 receptor | PEX5 | 3 | 0.631 | 0.319 | 0.5297 | 0.208 |
| 19 | IPI:IPI00032313.1 | S100A4 Protein S100-A4 | S100A4 | 8 | 0.6081 | 0.394 | 0.2168 | 0.251 |
| 20 | IPI:IPI01018712.1 | ACTA2 ACTA2 protein (Fragment) | ACTA2 | 70 | 0.5916 | 0.411 | 0.5495 | 0.372 |
| 21 | IPI:IPI00854743.1 | LOC652113 Immunglobulin heavy chain variable region | IGHV3-49 | 5 | 0.5861 | 0.515 | 0.207 | 0.322 |
| 22 | IPI:IPI01014928.1 | MAP1S cDNA FLJ55328, highly similar to Homo sapiens BPY2 interacting protein 1 (BPY2IP1), mRNA | MAP1S | 10 | 0.5598 | 0.529 | 0.5808 | 0.396 |
| 23 | IPI:IPI00968174.1 | IQGAP2 Uncharacterized protein | IQGAP2 | 20 | 0.5598 | 0.447 | 0.177 | 4E-04 |
| 24 | IPI:IPI00304273.2 | APOA4 Apolipoprotein A-IV | APOA4 | 11 | 0.5546 | 0.046 | 0.2704 | 0.008 |
| 25 | IPI:IPI00008529.1 | RPLP2 60S acidic ribosomal protein P2 | RPLP2 | 17 | 0.5445 | 0.092 | 0.5598 | 0.129 |
| 26 | IPI:IPI00465256.4 | AK3 GTP:AMP phosphotransferase, mitochondrial | AK3 | 7 | 0.5445 | 0.273 | 0.5546 | 0.124 |
| 27 | IPI:IPI00967716.1 | SEPT11 Uncharacterized protein | SEPT11 | 11 | 0.5395 | 0.269 | 0.2089 | 0.182 |
| 28 | IPI:IPI00221000.1 | BIN1 Isoform BIN1 of Myc box-dependent-interacting protein 1 | BIN1 | 6 | 0.5395 | 0.203 | 0.4246 | 0.133 |
| 29 | IPI:IPI00967977.2 | CD14 Conserved hypothetical protein | CD14 | 9 | 0.5346 | 0.521 | 0.52 | 0.103 |
| 30 | IPI:IPI00942356.1 | AKAP2 Isoform 5 of A-kinase anchor protein 2 | AKAP2 | 5 | 0.5346 | 0.083 | 0.4831 | 0.075 |
|  |  |  |  |  |  |  |  |  |

**The top 15 up-regulated proteins and top 15 down-regulated proteins in total were identified in abundance beyond the determined cut off (>1.3 or <0.7, P<0.05) across all the iTRAQ ratios. P values were calculated using the Paragon Algorithm in the ProteinPilot software.**
